# Value of 18F-Fluorodeoxyglucose (FDG) Positron Emission Tomography (PET)/Computed Tomography (CT) in the Detection of Recurrence in Medullary Thyroid Carcinoma With Negative Conventional Imaging: A Case Report

**DOI:** 10.7759/cureus.89326

**Published:** 2025-08-04

**Authors:** David Gutierrez Albenda, Julyana Murillo Jiménez, Laura Natalia Rodríguez Varela, Paula Ulate Blanco, Nelson Mauricio Sánchez Hidalgo

**Affiliations:** 1 Cyclotron-PET/CT Laboratory, University of Costa Rica, San José, CRI; 2 School of Medicine, University of Costa Rica, San José, CRI

**Keywords:** 18f-fdg, medullary thyroid carcinoma, metastasis, nuclear medicine, positron emission tomography/computed tomography (pet/ct), recurrence

## Abstract

Medullary thyroid carcinoma (MTC) is a rare neuroendocrine tumor with variable clinical presentation, posing both diagnostic and therapeutic challenges. Disease recurrence is common and may manifest solely as elevated tumor markers in the absence of clinical findings or positive morphological imaging. We present the case of a 56-year-old woman diagnosed with MTC in 2011, treated with total thyroidectomy and adjuvant therapy. In 2021, a locoregional recurrence was detected, requiring left-sided neck dissection. During follow-up in 2025, elevated tumor markers were identified despite negative findings on computed tomography (CT). Consequently, an ^18^F-fluorodeoxyglucose (FDG) positron emission tomography (PET)/CT was performed, revealing disease recurrence with multiple lesions suggestive of bone metastases. This case underscores the value of ^18^F-FDG PET/CT in the follow-up and detection of MTC recurrence.

## Introduction

Medullary thyroid carcinoma (MTC) is a rare neuroendocrine tumor, accounting for approximately 5% of all thyroid cancers, and arises from parafollicular C cells that produce calcitonin [1,2]. It occurs more frequently in sporadic form (75%) and, less commonly, as part of hereditary syndromes such as multiple endocrine neoplasia type 2 (MEN2) (25%) [1,3].

MTC is often diagnosed at advanced stages, with lymph node metastases present in up to 60% of cases at diagnosis. It tends to follow an unusual pattern of spread, with micrometastases that are difficult to detect with conventional imaging. The disease course can be variable and may progress rapidly even after prolonged periods of apparent stability [1-5].

Initial treatment of MTC is surgical. However, in patients with distant metastases, therapeutic options are limited due to the poor response to chemotherapy and radiotherapy [1,5]. In such patients, both calcitonin and carcinoembryonic antigen (CEA), particularly their doubling times (DT)-defined as the time required for their serum concentration to double-are crucial as markers of disease monitoring and progression. Shorter DT correlates with more aggressive tumor behavior, advanced disease progression, and unfavorable survival rates [1,2,4].

Conventional morphological imaging techniques, such as ultrasound (US), computed tomography (CT), and magnetic resonance imaging (MRI), detect approximately 40% of recurrences suspected based on laboratory findings [6].

In this context, hybrid imaging such as 18F-fluorodeoxyglucose (18F-FDG) positron emission tomography (PET)/CT, which provides both functional imaging (metabolic activity of tissue) and morphological imaging (structural detail) in a single study, has demonstrated higher sensitivity for lesion detection. This is particularly relevant in cases with biochemical evidence of disease (e.g., elevated calcitonin or CEA) without radiological correlation, a condition often referred to as biochemical-radiological discordance, where occult or micrometastatic disease is suspected and advanced imaging is required to accurately identify metastatic foci [3,5].

This article presents the case of a patient with MTC who exhibited persistent elevation of tumor markers with no clinical signs or abnormalities on conventional imaging. An 18F-FDG PET/CT revealed locoregional recurrence and bone metastases. This case highlights the importance of nuclear medicine imaging, such as 18F-FDG PET/CT, particularly in the evaluation of MTC recurrence.

## Case presentation

A 56-year-old female patient was diagnosed with stage III MTC in 2011. In 2012, she underwent total thyroidectomy followed by radiotherapy and six cycles of adjuvant chemotherapy. Subsequently, she received treatment with sorafenib for one year and continued under strict surveillance.

In 2021, left cervical lymph node recurrence was documented, for which a left neck dissection was performed the same year. The patient remained under regular follow-up in the oncology department without clinical or radiological evidence of tumor recurrence.

However, in April 2025, elevated levels of serum CEA at 17.9 ng/mL (normal range: 2.5 - 5 ng/mL) and serum calcitonin at 185 pg/mL (post-thyroidectomy reference: undetectable or <2 pg/mL) were observed, raising concern for disease recurrence, despite the patient remaining clinically asymptomatic at that time.

A contrast-enhanced CT scan of the chest, abdomen, and pelvis was performed during the same period, which revealed no lesions suggestive of recurrence. Given the biochemical-radiological discordance, the patient was referred to our center for a more detailed evaluation with 18F-FDG PET/CT. At the time of referral, no prior tumor marker data were provided by the treating physician, which limited the evaluation of long-term biochemical trends. In June 2025, a PET/CT scan was performed 60 minutes after intravenous administration of 9.4 mCi (348 MBq) of 18F-FDG. The scan covered from the skull vertex to the mid-thigh using a high-resolution PET/CT system. Low-dose CT was performed on a 128-slice helical multidetector scanner.

**Figure 1:**
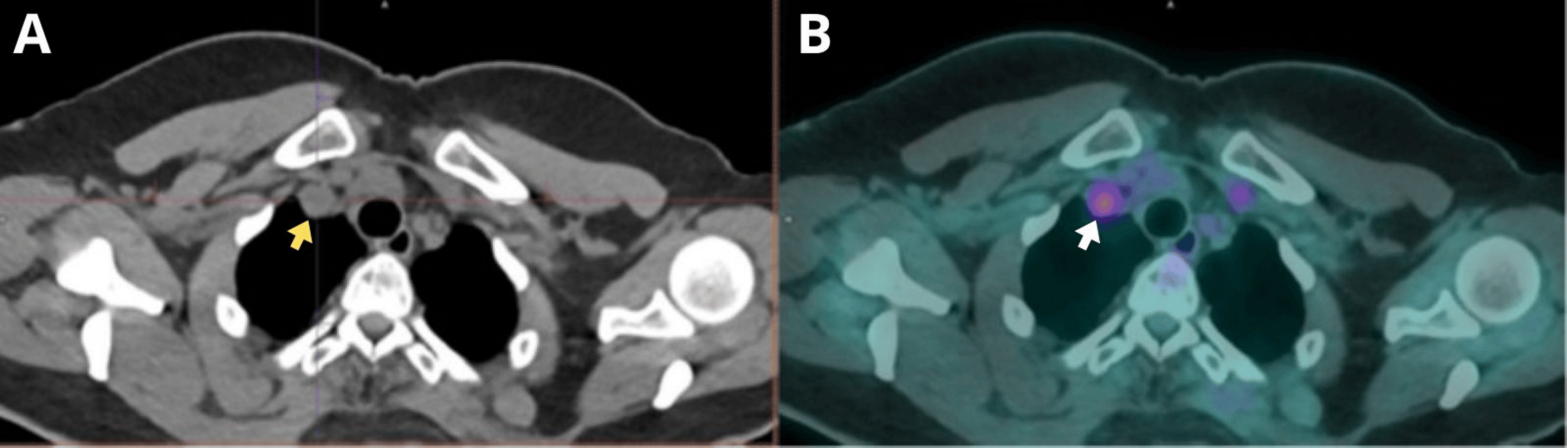
A) Axial CT scan showing a lymph node in the right level IV cervical region, measuring 1.1 cm (yellow arrow). B) Axial fused PET/CT image demonstrating hypermetabolism in the cervical lymph node, SUVmax of 4.1 (white arrow). CT: computed tomography; PET/CT: positron emission tomography/computed tomography

**Figure 2:**
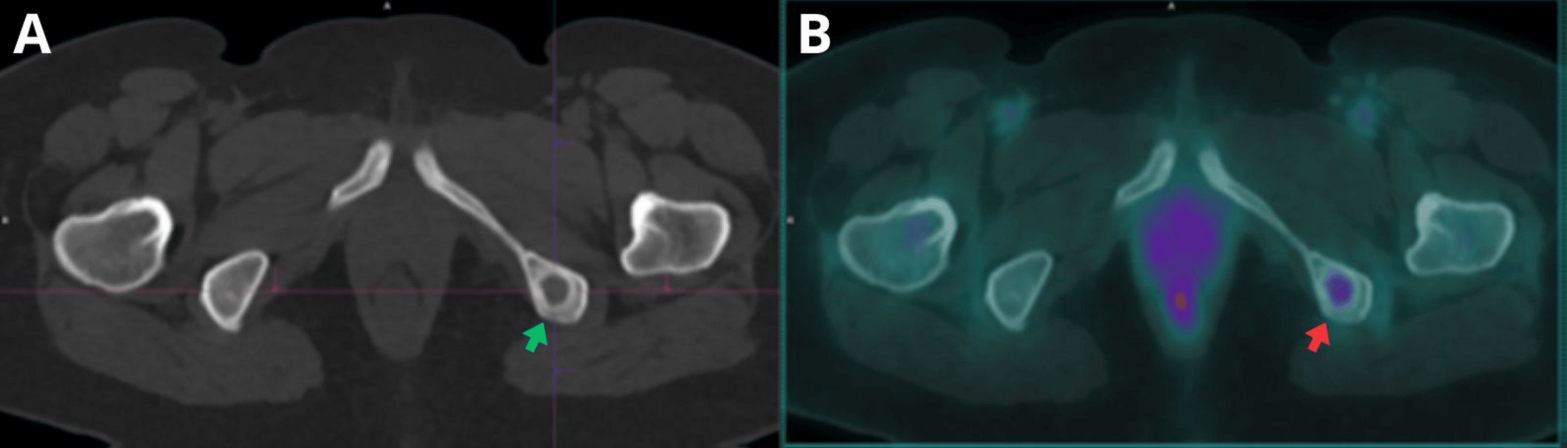
A) Axial CT scan showing an osteolytic lesion in the left ischium, measuring 1.4 cm (green arrow). B) Axial fused PET/CT image demonstrating hypermetabolism in the osteolytic lesion, SUVmax of 2.0 (red arrow). CT: computed tomography; PET/CT: positron emission tomography/computed tomography

**Figure 3:**
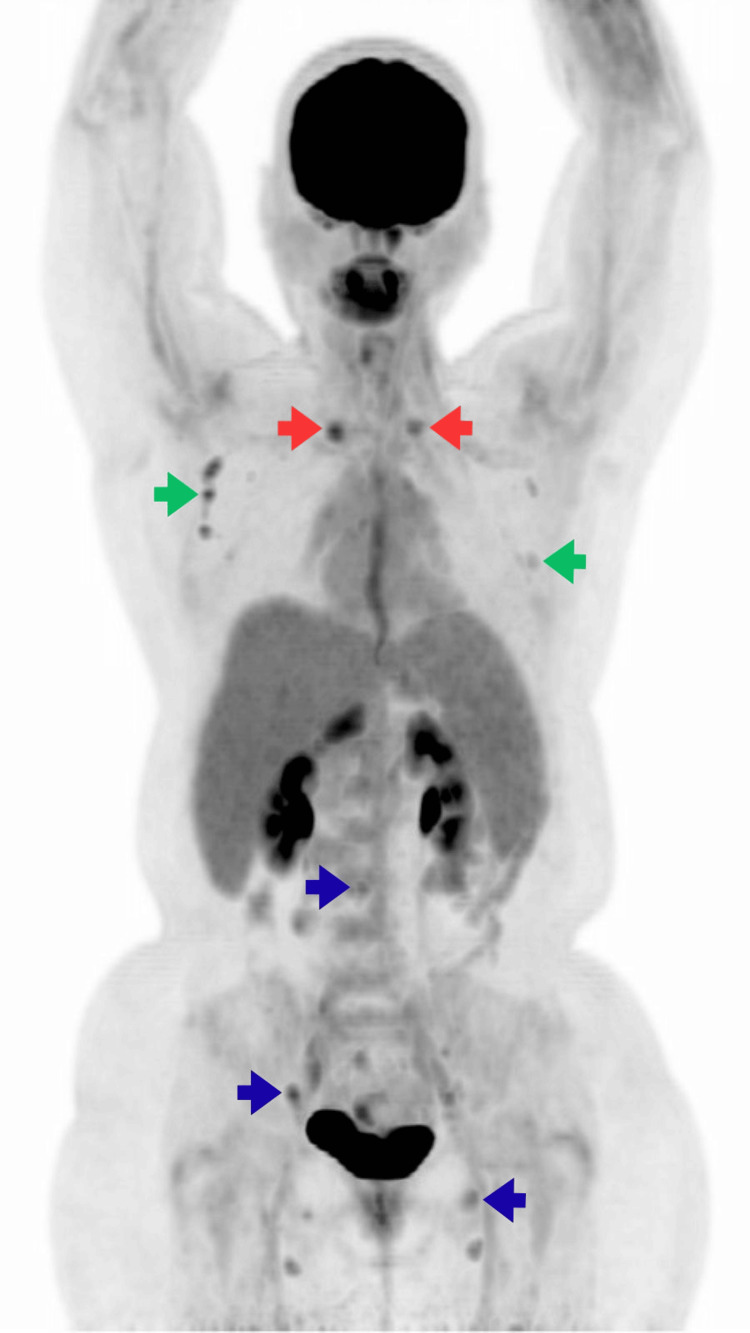
Maximum intensity projection (MIP) of the whole-body ¹⁸F-FDG PET/CT. Hypermetabolic cervical lymph nodes (red arrow) and osseous lesions are visualized (blue arrow). Bilateral axillary lymph nodes with a reactive appearance are also noted (green arrow). ¹⁸F-FDG: 18F-fluorodeoxyglucose; PET/CT: positron emission tomography/computed tomography

The study revealed multiple sites of abnormal FDG uptake consistent with disease recurrence and dissemination. Bilateral low cervical and supraclavicular lymphadenopathies were noted, the largest measuring 1.1 cm in short-axis diameter with an SUVmax of 4.1 on the right side, suggestive of neoplastic infiltration (Figures 1-3). Multiple hypermetabolic bone lesions were identified throughout the axial and appendicular skeleton, including the vertebral column, sacrum, pelvic bones, and right proximal femur. Most lesions had well-defined sclerotic borders; the largest was located in the left ischium, measuring 1.4 cm with an SUVmax of 2.0, consistent with metastatic bone disease (Figures 2-3).

## Discussion

MTC presents a clinical and diagnostic challenge due to its low incidence, high metastatic potential, and variable progression. The most common clinical presentation is a palpable cervical mass, sometimes associated with compressive symptoms such as dysphagia, pain, dyspnea, or dysphonia; however, it may also be asymptomatic. The presence of systemic symptoms such as diarrhea or facial flushing usually indicates advanced or metastatic disease [1, 5, 7].

Regarding diagnosis, a wide range of biochemical, radiological, and genetic methods is available. Initial approaches include neck US and fine needle aspiration biopsy. Studies have shown that the US has limited performance in identifying MTC, as its sonographic features may resemble those of well-differentiated thyroid carcinoma, potentially leading to delayed diagnosis and worse prognosis [5, 7, 8].

Biochemically, serum calcitonin, secreted specifically by parafollicular C cells, is a highly sensitive biomarker for the diagnosis and postoperative monitoring of MTC. CEA is a nonspecific tumor marker used as a complementary tumor marker, particularly useful in monitoring disease progression and recurrence when interpreted alongside calcitonin [1, 2, 4].

In particular, the evaluation of the DT of these markers has demonstrated strong prognostic value and is considered an indicator of disease progression. A DT shorter than 24 months is associated with aggressive tumor behavior, advanced disease, and reduced overall survival [1,5,8].

The treatment offering the highest likelihood of cure in MTC is complete surgical resection of the tumor with lymph node dissection [8]. However, in patients with distant metastases, the prognosis worsens significantly due to the poor response to chemotherapy and radiotherapy, underscoring the importance of timely diagnosis and management [1,5,8].

Even after surgical treatment, local recurrence and metastatic disease are common, which is why comprehensive clinical follow-up is recommended, including annual biochemical and radiological assessment with calcitonin and CEA [5,9]. Postoperative normalization of calcitonin levels is considered the most significant prognostic factor [8]. Early detection of recurrence in MTC is challenging; however, persistent elevation of these markers may indicate residual or recurrent disease, in which case advanced imaging is necessary to identify metastatic foci [1,5,9].

Imaging plays a fundamental role in both initial diagnosis and the follow-up and evaluation of recurrent disease. Conventional morphological imaging modalities such as US, CT, and MRI are used for preoperative staging. Patients with preoperative calcitonin levels >500 pg/mL or postoperative levels >150 pg/mL require more thorough radiological stratification due to the high suspicion of disseminated disease [9-11].

Standard morphological imaging has limited sensitivity, detecting up to 40% of recurrences suspected based on biochemical findings [6]. This scenario, often seen in biochemical-radiological discordance, likely reflects occult or micrometastatic disease that conventional scans fail to detect [11,12]. In such cases, functional imaging such as PET/CT is essential, as it can detect metabolic alterations that may precede any structural changes detectable on a conventional scan. Although its role is more restricted in the preoperative setting, PET/CT provides valuable information for identifying and localizing recurrent disease [10-12].

Various radiopharmaceutical imaging modalities exist, but PET/CT with ^18^F-fluorodeoxyglucose (^18^F-FDG), fluorine-18 dihydroxyphenylalanine (^18^F-FDOPA), and gallium-68 somatostatin analogs (68Ga-SSA) are the most sensitive [1,5,11].

^18^F-FDG is currently the most widely used radiopharmaceutical in oncological PET imaging. As a glucose analog, it detects tumors with increased glucose metabolism. ^18^F-FDG uptake correlates with high proliferative activity, low cellular differentiation, and decreased survival, making it an effective technique for predicting overall patient survival [6,5,11,13].

^18^F-FDG PET/CT is a whole-body imaging modality that enables a detailed characterization of occult lesions, typically requested following negative conventional imaging [8,13]

A positive correlation exists between tumor marker levels and the sensitivity of ^18^F-FDG PET/CT. Notably, its sensitivity increases with higher serum calcitonin and CEA levels and shorter DT [8,11,12]. The combined assessment of ¹⁸F-FDG PET/CT findings and calcitonin DT further enhances the stratification of high-risk patients, for whom close surveillance or earlier therapeutic interventions may be warranted [5,12,13].

Beyond its diagnostic accuracy, ¹⁸F-FDG PET/CT findings can directly influence clinical decision-making, including consideration of systemic therapies such as tyrosine kinase inhibitors, radioimmunotherapy, or palliative interventions for symptomatic metastases [5,6]. Additionally, emerging evidence suggests that FDG uptake patterns in PET/CT may have prognostic implications, as certain metastatic distributions and intensity profiles have been linked to more aggressive disease biology in MTC [14].

This dual diagnostic and prognostic capability is particularly relevant in advanced or rapidly progressive MTC, where early and accurate detection of recurrence can guide tailored management strategies [5,9,12,13].

In cases of more aggressive disease, such as the one reported in this article - a patient with MTC who underwent multimodal treatment, presented with elevated tumor markers, negative morphological and clinical suspicion of recurrence - ^18^F-FDG PET/CT is the imaging modality of choice [13].

## Conclusions

This case highlights the value of ¹⁸F-FDG PET/CT in evaluating suspected recurrence in patients with MTC, particularly when serum tumor markers are elevated despite negative findings on conventional imaging. In this patient, ¹⁸F-FDG PET/CT revealed bilateral hypermetabolic cervical lymph nodes and multiple lytic bone lesions consistent with metastatic spread, none of which were detected by prior CT. These findings provided essential information that confirmed recurrence, defined the disease extent, and guided subsequent clinical decision-making. This case reinforces the complementary and often decisive role of functional imaging in complex oncologic scenarios, supporting its inclusion in the diagnostic pathway for advanced MTC.
